# Why Lyme disease is common in the northern US, but rare in the south: The roles of host choice, host-seeking behavior, and tick density

**DOI:** 10.1371/journal.pbio.3001066

**Published:** 2021-01-28

**Authors:** Howard S. Ginsberg, Graham J. Hickling, Russell L. Burke, Nicholas H. Ogden, Lorenza Beati, Roger A. LeBrun, Isis M. Arsnoe, Richard Gerhold, Seungeun Han, Kaetlyn Jackson, Lauren Maestas, Teresa Moody, Genevieve Pang, Breann Ross, Eric L. Rulison, Jean I. Tsao

**Affiliations:** 1 US Geological Survey, Patuxent Wildlife Research Center, Woodward-PSE, University of Rhode Island, Kingston, Rhode Island, United States of America; 2 Department of Plant Sciences and Entomology, University of Rhode Island, Kingston, Rhode Island, United States of America; 3 Center for Wildlife Health, University of Tennessee Institute of Agriculture, Knoxville, Tennessee, United States of America; 4 Department of Biology, Hofstra University, Hempstead, New York, United States of America; 5 Public Health Risk Sciences Division, National Microbiology Laboratory, Public Health Agency of Canada, Ste-Hyacinthe, Quebec, Canada; 6 US National Tick Collection, Institute for Coastal Plain Science, Georgia Southern University, Statesboro, Georgia, United States of America; 7 Department of Fisheries and Wildlife, Michigan State University, East Lansing, Michigan, United States of America; 8 Comparative Medicine and Integrative Biology, Michigan State University, East Lansing, Michigan, United States of America; Princeton University, UNITED STATES

## Abstract

Lyme disease is common in the northeastern United States, but rare in the southeast, even though the tick vector is found in both regions. Infection prevalence of Lyme spirochetes in host-seeking ticks, an important component to the risk of Lyme disease, is also high in the northeast and northern midwest, but declines sharply in the south. As ticks must acquire Lyme spirochetes from infected vertebrate hosts, the role of wildlife species composition on Lyme disease risk has been a topic of lively academic discussion. We compared tick–vertebrate host interactions using standardized sampling methods among 8 sites scattered throughout the eastern US. Geographical trends in diversity of tick hosts are gradual and do not match the sharp decline in prevalence at southern sites, but tick–host associations show a clear shift from mammals in the north to reptiles in the south. Tick infection prevalence declines north to south largely because of high tick infestation of efficient spirochete reservoir hosts (rodents and shrews) in the north but not in the south. Minimal infestation of small mammals in the south results from strong selective attachment to lizards such as skinks (which are inefficient reservoirs for Lyme spirochetes) in the southern states. Selective host choice, along with latitudinal differences in tick host-seeking behavior and variations in tick densities, explains the geographic pattern of Lyme disease in the eastern US.

## Abstract 2

Lyme disease is common in the northeastern United States, but rare in the southeast, even though the tick vector is found in both regions. This study shows that this is largely because the tick vectors attach abundantly to rodents (which are good hosts for the Lyme bacteria) in the north, and to lizards (which are relatively poor hosts for Lyme bacteria) in the south.

## Introduction

Lyme disease is the most common vector-borne disease in North America, with an estimated 300,000 cases annually in the United States [1]. A particular observation about Lyme disease distribution has been noted by numerous investigators and has also resulted in confusion about the geographic risk of Lyme disease for the public: The distribution of the tick vector does not match the distribution of human cases. The major vector of Lyme disease in North America is the blacklegged tick, *Ixodes scapularis*, which is abundant in the northeastern and northcentral US, extending south into the Gulf states from Texas to Florida [2]. Human cases of Lyme disease, however, are concentrated in the northern part of the range, with relatively few cases in the south [3]. This reflects an enduring question about the geographical distributions of many vector-borne diseases: What factors underlie the relationships among environmental conditions, vector and pathogen distributions, and human disease risk?

Several hypotheses have been proposed to explain this phenomenon, generally related to either tick host-seeking behavior or to tick–host associations. North–south differences in tick host-seeking behavior can affect the number of tick bites, potentially resulting in fewer humans being bitten in the south [4,5]. The community composition of vertebrate host species also differs north to south [6], and this variation could profoundly affect pathogen transmission patterns. The possible ecological effect of alternative host species lowering transmission of vector-borne pathogens to humans has been called “zooprophylaxis,” which can result from alternative host species diverting vectors from humans [7], or from diversion of ticks from reservoir hosts in zoonoses such as Lyme disease [8]. For zoonotic pathogens, this phenomenon could potentially take 2 forms, which are not mutually exclusive. One is a dilution [9] or buffering [10] effect in which increased host diversity results in the distribution of ticks among diverse hosts, lowering the numbers of ticks on species that are highly competent reservoir hosts. In contrast, ticks might selectively attach to hosts that are poor reservoirs, which is not dilution, but rather host selection. In the case of Lyme disease, a primary reservoir host in the northeastern US is the white-footed mouse, *Peromyscus leucopus* [11], and increased host diversity could result in more ticks on hosts that are comparatively poor reservoirs of the Lyme spirochete, *Borrelia burgdorferi* sensu stricto, such as opposums (*Didelphis virginianus*) and raccoons (*Procyon lotor*) (see Table 1). The dilution effect might manifest in terms of the clinal geographical distribution of Lyme disease, because vertebrate diversity (in particular, of mammals and reptiles) increases from north to south, so that southern ticks are distributed on numerous host species in addition to mice. This, in turn, might result in fewer ticks on mice in the south and therefore, in lower *B*. *burgdorferi* infection prevalence in questing ticks [6]. The alternative hypothesis cites specific host associations of northern versus southern ticks and argues that southern ticks are particularly abundant on lizards, which are particularly poor reservoirs of Lyme spirochetes [12,13]. In this view, it is not dilution of generalist ticks among hosts, but rather specific host selection patterns that result in lower prevalence of Lyme disease in the south.

**Table 1:** Reservoir competence of species in different categories of tick hosts.

Host category	Species	Reservoir competence (%)	Reference
Mice	*Peromyscus leucopus*	Approximately 75^a^	[21]
		89^b^	[10]
	*Peromyscus maniculatus*	80^a^	[22]
Voles	*Microtus pennsylvanicus*	1–6^b^	[23]
		Approximately 62^b^–84^a^	[24]
Shrews	*Sorex* spp.	51^b^	[25]
	*Blarina brevicauda*	37^b^	[26]
		42^b^	[25]
Squirrels and rats	*Tamias striatus*	20^b^	[10]
		55^b^	[25]
	*Sciurus carolinensis*	17^b^	[23]
		15^b^	[25]
	*Oryzomys palustris*	76^a^	[27]
	*Rattus norvegicus*	72^a^	[28]
Medium mammals	*Procyon lotor*	14^b^	[29]
		0^b^	[23]
		1^b^	[25]
	*Didelphis virginianus*	3^b^	[25]
Skinks	*Plestiodon inexpectatus*	24^a^	[30]
	*Plestiodon* spp.	0.005^a^	[31]*
Other lizards	*Sceloporus undulatus*	0^a^	[32]*
	*Anolis carolinensis*	2^a^	[30]

^a^Lab study: xenodiagnoses using larvae placed on previously infected animals in lab.

^b^Field study: infection in nymphs from larvae collected from animals in the field.

*Research done as part of the current study.

Despite the important ecological implications of these 2 hypotheses, and the implications for disease management, north–south trends in tick–host associations have not previously been studied using standardized methods over broad enough geographical areas to allow comparison of these hypotheses. We report the results of a broad geographic study that characterizes host associations of ticks based on 8 sample sites, each of which underwent intensive sampling of ticks, hosts, and environmental factors, including habitat characteristics and physical factors. These sites were selected to represent appropriate habitat for *I*. *scapularis* throughout its range. They consisted of forest habitats with canopy, shrub layers (appropriate for adult questing), and leaf litter suitable for larval and nymphal habitat. Each site had 2 to 3 sampling arrays, spaced >3-km apart (to minimize movement of hosts among arrays). Each array was approximately1 ha, including live traps for all ground-dwelling vertebrates, including Sherman and Tomahawk traps, pitfall trap arrays, wood and metal cover boards, burlap skirts on trees, motion-activated wildlife cameras, and flag/drag transects for free-living ticks, with a weather station to record temperature and relative humidity. Sample sites were located throughout the eastern US, in Wisconsin, Massachusetts, New Jersey, North Carolina, Tennessee, South Carolina, Alabama, and Florida, all in woodland areas with appropriate habitat for the tick vector, *I*. *scapularis*. A sample site in Rhode Island was added for flag/drag samples and mouse samples to collect additional spirochete prevalence and mouse infestation data. Tick life history patterns, along with physical and ecological characteristics of these sites, have previously been reported [14–16].

## Results

### Latitudinal trends in tick infection with Lyme spirochetes

**Fig 1:**
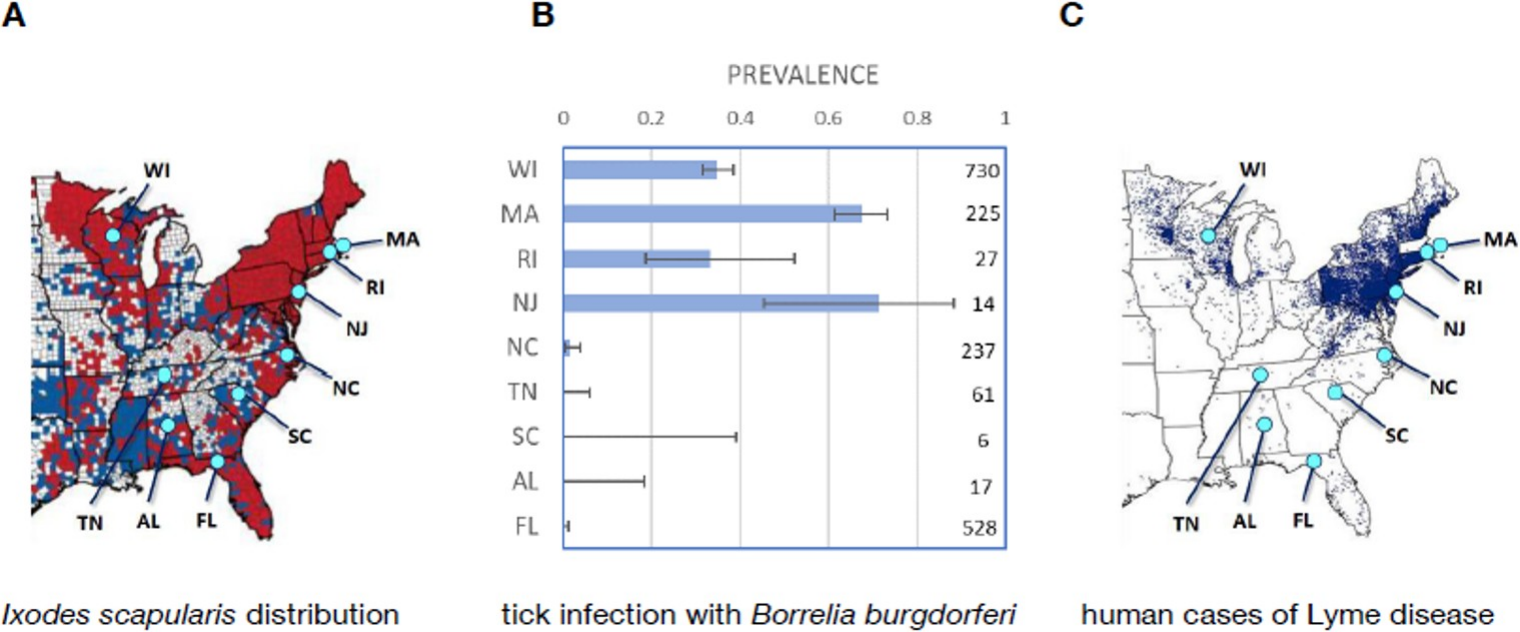
Locations of study sites and tick infection prevalence. (**A**) Map of distribution of *Ixodes scapularis* ticks; red counties have tick populations that are considered established (at least 6 ticks or at least 2 of the host-seeking life stages had been identified in a single collection period), and blue counties have at least some tick collections, but establishment has not been demonstrated [2]. (**B**) Infection prevalence in host-seeking adult *I*. *scapularis* with binomial 95% confidence intervals (from current study; number to the right of each bar is the number of ticks tested to provide that infection prevalence value). (**C**) Map of human cases of Lyme disease in the eastern and central US in 2018 (CDC map; each dot indicates 1 case of Lyme disease, placed randomly in the patient’s county of residence; https://www.cdc.gov/lyme/stats/maps.html). Maps in Fig 1A and 1C are from the CDC, and data in Fig 1B are available in S1 Data. CDC, Centers for Disease Control and Prevention.

Infection of host-seeking ticks with Lyme spirochetes, *B*. *burgdorferi*, declined from north to south, with a precipitous drop below the latitude of the state of Virginia (Fig 1). The locations of our sample sites are shown in relation to the geographical distributions of *I*. *scapularis* in Fig 1A, infection prevalence of host-seeking adult ticks at these sites is shown in Fig 1B, and human cases of Lyme disease in Fig 1C. Free-living nymphs are difficult to collect in the southern US (S1 Fig; data are available in S2 Data) because southern nymphs rarely quest above the leaf litter [17], so we report infection prevalence of host-seeking adult ticks in this figure (a total of 14 free-living nymphs were collected and tested from the southern sites; all were negative). Infection prevalence differed between northern and southern sites (logistic regression, Wald ***χ***^***2***^ = 18.606, df = 1, ***p*** < 0.0001), and there was a significant interaction between the effect of latitude and north/south location (Wald ***χ***^***2***^ = 11.257, df = 1, ***p*** = 0.0008), so that when the effect of north/south location was included in the model, there was no further effect of latitude on prevalence (Wald ***χ***^***2***^ = 0.256, df = 1, ***p*** = 0.613). These results agree with other investigators, such as Xu and colleagues [18], who recently reported a precipitous decline in infection of host-seeking adult *I*. *scapularis* at about the same latitude. The reduced incidence of human Lyme disease cases along a north–south gradient (Fig 1C) shows a direct relationship to the spirochete prevalence in ticks and is characterized by a precipitous drop in both tick infection and human disease south of about 37° N latitude.

### Latitudinal trends in host species diversity and tick–host associations

**Fig 2:**
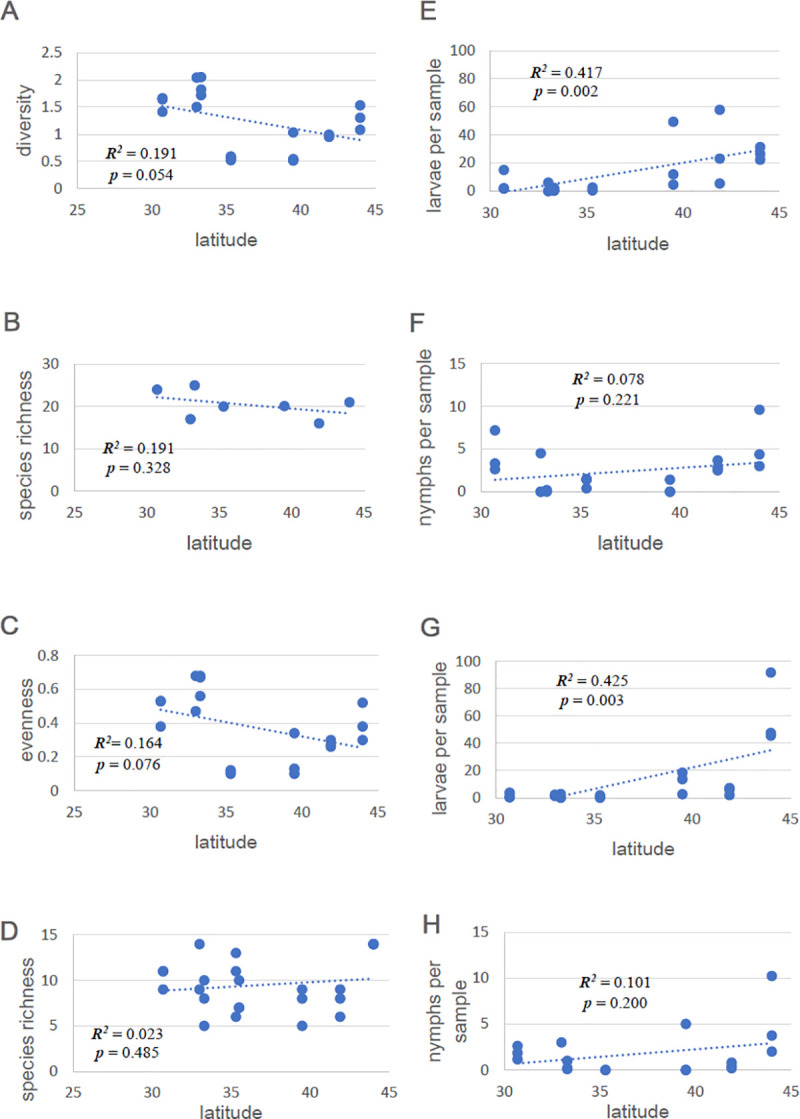
Latitudinal trends in host diversity and tick abundance. Species diversity of mammals + reptiles at field sites as a function of latitude. (**A**) Shannon–Wiener Index (H’) for trapped species at each sampling array. (**B**) Species richness (overall for each of 7 sites; estimated total number of species from trap data, using SPECRICH). (**C**) Evenness (1-BP) of species captured in traps at each sampling array. (**D**) Species richness (number of species) of animals photographed by remote cameras at each sampling array. Latitudinal trends in tick abundance (mean numbers of ticks on all hosts collected in each sampling array) of (**E**) larvae in 2011, (**F**) nymphs in 2011, (**G**) larvae in 2012, and (**H**) nymphs in 2012. The Rhode Island site used only mouse traps and vertebrate trapping was limited at the North Carolina site (no pitfall traps), so these sites are not included in the figures or the accompanying analyses. Data for Fig 2A–2D are available in S3 Data. Data for Fig 2E–2H are available in S4 Data.

Ground-dwelling mammals and reptiles at our sample sites, those that are most likely to be encountered by ticks, did not show strong latitudinal trends in diversity (Fig 2A–2D). Trends among captured animals in species diversity (Shannon–Wiener Index, Fig 2A), species richness based on trap captures (estimated total number of species present at each of the 7 sites with full samples, using SPECRICH, https://www.mbr-pwrc.usgs.gov/software/specrich.html, Fig 2B), and evenness (= 1 − Berger–Parker Index, Fig 2C) [19] showed evidence of modest decline from south to north, but these trends were not statistically significant. The numbers of medium and large vertebrate species seen in camera traps (Fig 2D) showed no decline with latitude. Our sampling protocols were the same among sites, so rather than using estimators of species richness, one could use the total numbers of species in the actual samples to avoid assumptions of estimators that might apply differently at different sites. Total numbers of species captured in each array (***n*** = 20 arrays) showed a similar trend to that based on estimated total numbers of species present, with an apparent but modest negative trend south to north that was not statistically significant (***R***^***2***^ = 0.108, ***p*** = 0.157). These trends are for overall species richness at these sites. We can fine-tune the analysis by examining trends within each year at each of the 7 sites for which we had full samples and by including only species groups that ticks used as hosts (mammals and lizards) and only at the times of year when larvae and nymphs were active (S1 Table). Using this approach, trends with latitude were not statistically significant in 2011 for Shannon–Wiener diversity (***R***^***2***^ = 0.114, ***p*** = 0.145), species richness (***R***^***2***^ = 0.0039, ***p*** = 0.794), or evenness (***R***^***2***^ = 0.176, ***p*** = 0.065). In 2012, there were modest but significant trends with latitude for diversity (***R***^***2***^ = 0.283, ***p*** = 0.019) and evenness (***R***^***2***^ = 0.262, ***p*** = 0.025), but not for species richness (***R***^***2***^ = 0.171, ***p*** = 0.079). Clearly, species diversity alone does not provide a good match to trends in tick infection prevalence (Fig 1B) and does not appear to explain the rapid drop in infection prevalence below the 37th parallel.

**Fig 3:**
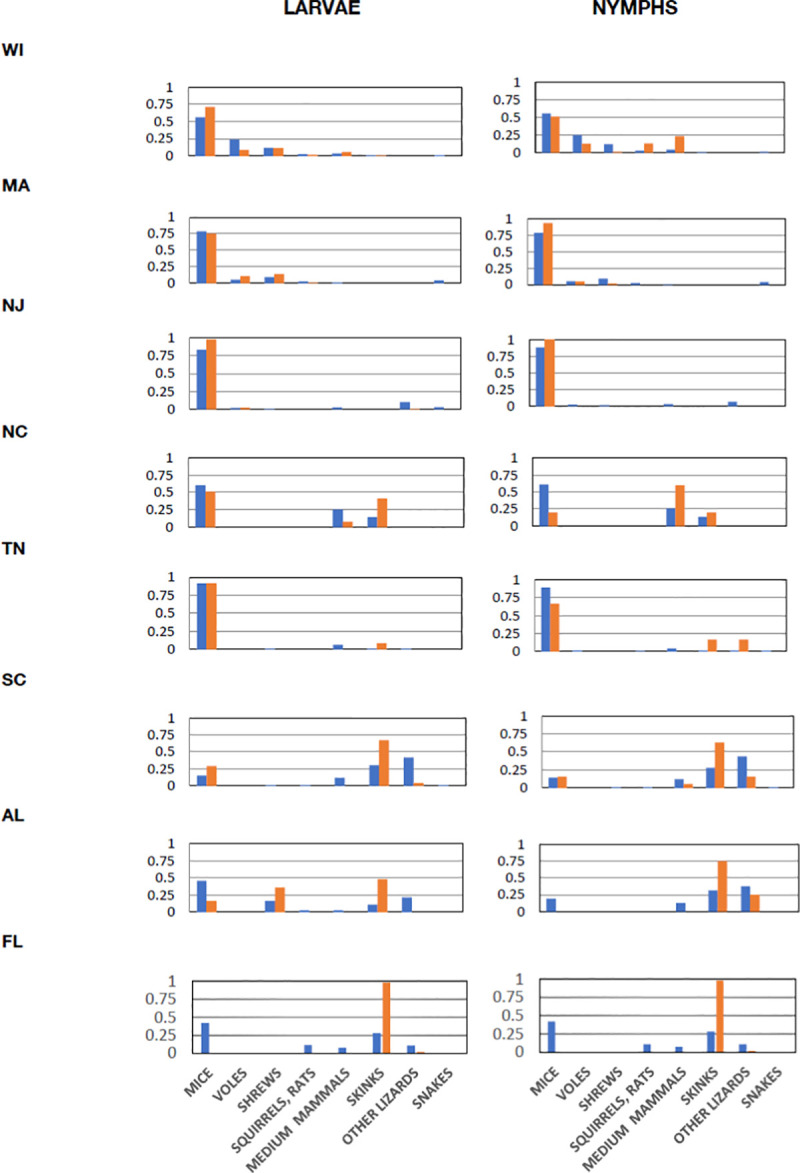
Proportions of hosts of different categories in samples and proportions of ticks collected from hosts in each category. Blue bars are proportions of hosts in each category, and orange bars are proportions of ticks collected from hosts in each category. Taxa in each category: mice (*Peromyscus* and *Ochrotomys*), voles (*Myodes* and *Microtus*), shrews (*Sorex* and *Blarina*), squirrels, rats (*Tamias*, *Glaucomys*, *Tamiasciurus*, *Neotoma*, and *Oryzomys*), medium mammals (*Procyon* and *Didelphis*), skinks (*Plestiodon* and *Scincella*), other lizards (*Sceloporus* and *Anolis*), and snakes (*Diadophis*, *Storeria*, *Thamnophis*, and *Coluber*). Data combined from 2011 and 2012 (when all 8 sites were sampled). Data are available in S5 Data (raw data in S3 Data).

**Fig 4:**
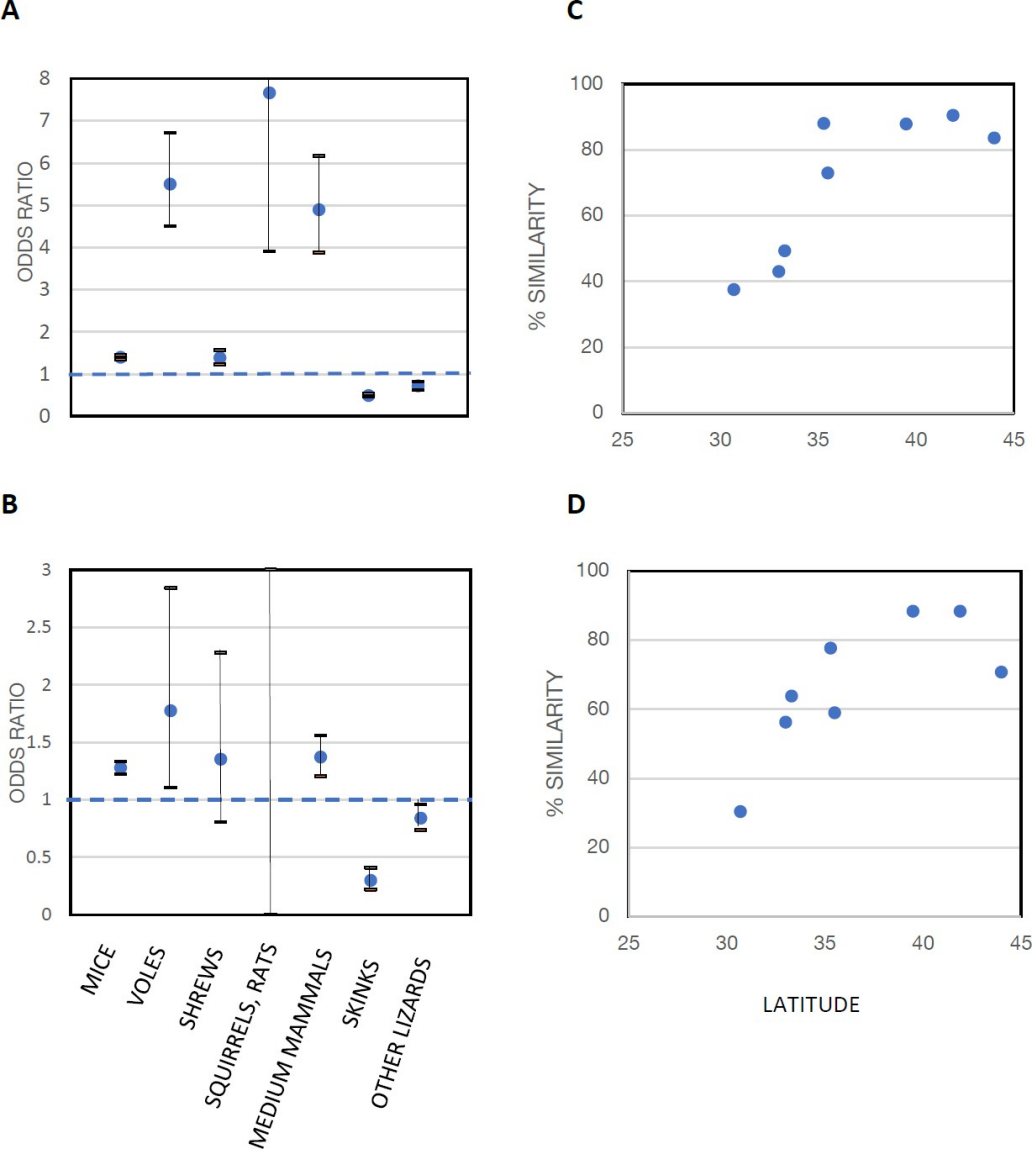
Latitudinal trends in tick–host associations. (**A, B**) Logistic regression of changes in proportion of (A) larvae and (B) nymphs from hosts in each category with latitude. Points are odds ratios with 95% Wald confidence limits. (**C, D**) Latitudinal patterns of percent similarity of distributions of individual animals sampled from each host category with distributions of (C) larvae and (D) nymphs collected from hosts in those categories. Data for Fig 4A and 4B are available in S6 Data. Data for Fig 4C and 4D are available in S7 Data.

To assess tick–host relationships at our sites, we divided vertebrate species into 8 categories: (1) mice; (2) voles; (3) shrews; (4) squirrels and rats (chipmunks, squirrels and flying squirrels, woodrats, etc.); (5) medium-sized mammals (mostly raccoons and opossums); (6) skinks; (7) other lizards (mostly fence lizards and anoles); and (8) snakes. These categories were defined based on animal size and on estimates from previous literature of the relative importance of these groups as reservoirs for *B*. *burgdorferi* (see Materials and methods). The blue bars in Fig 3 are the proportions of animals in each category captured at each site, while the orange bars are the proportions of ticks removed from hosts from each category. Note that shrews were undersampled at the North Carolina site because of the lack of pitfall traps, but shrews were rarely collected at the other southern sites, all of which had pitfall traps. A clear north–south trend is evident, with northern ticks collected mostly from mammals, and increasing numbers of ticks collected from lizards at the southern sites. Fig 4A and 4B show odds ratios based on ticks collected from each category of hosts with latitude, north to south. Proportions of ticks collected from each category of hosts in our samples were significantly higher on small mammals at northern sites and on lizards at southern sites. If this resulted from simple dilution of ticks on more diverse hosts in the south, the similarity of the distributions of hosts among categories and of ticks from hosts in those categories would show no trend with latitude. We assessed this trend using percent similarity, which quantifies the similarity between 2 samples in the distributions of individuals among categories [20]. Percent similarities were lowest at the southern sites (Fig 4C and 4D), likely resulting from selective attachment to lizards in the south (Fig 3).

These host associations are important because different host species differ in reservoir competence for *B*. *burgdorferi*, the Lyme disease spirochete. Here, we define reservoir competence as the proportion of larval ticks that acquire infection after feeding on an infected host. Table 1 displays several literature estimates of reservoir competence of animals in our host categories. We did not include snakes because no *Ixodes* ticks were found on snakes in our samples (with the exception of 1 nymph on a smooth green snake in Wisconsin). Estimates of reservoir competence were based on lab studies (a) in which the host animals were infected in the lab (generally by feeding of infected nymphs) before uninfected larvae were allowed to feed on the animals; and on field studies (b), which generally involved placing larvae on field-caught hosts or collecting attached larvae dropping from these hosts (so it was unknown whether the host animals were infected before larval feeding). Infection status was tested after the larvae had engorged, dropped off, and molted to the nymphal stage. Differences among host categories are clear: Mice, voles, and shrews tend to be excellent reservoirs for *B*. *burgdorferi*, while larger mammals and lizards are not. Therefore, the preponderance of attachment to mice by larvae and nymphs in northern sites (Figs 3 and 4) results in efficient transmission of *B*. *burgdorferi* in ticks attaching to these highly competent reservoir species, while ticks in the south attach largely to lizards, which generally do not maintain spirochetal infection.

One problem, however, is that the different trapping methods we utilized are not equally effective at capturing animals from the various categories of hosts. As such, the values in Fig 3 show captures, but do not represent the actual proportions of host animals or of ticks present on each category of hosts at the site. This problem is not likely to affect geographical trends because the sampling protocol was the same among sites. However, these results quantify only the proportions of hosts collected from each host category and do not quantify tick abundance at the various sites. Abundance can be critical, because higher tick abundance can result in more ticks per individual host, which can affect the probability of spirochete transmission to and from that host animal [10,33].

**Fig 5:**
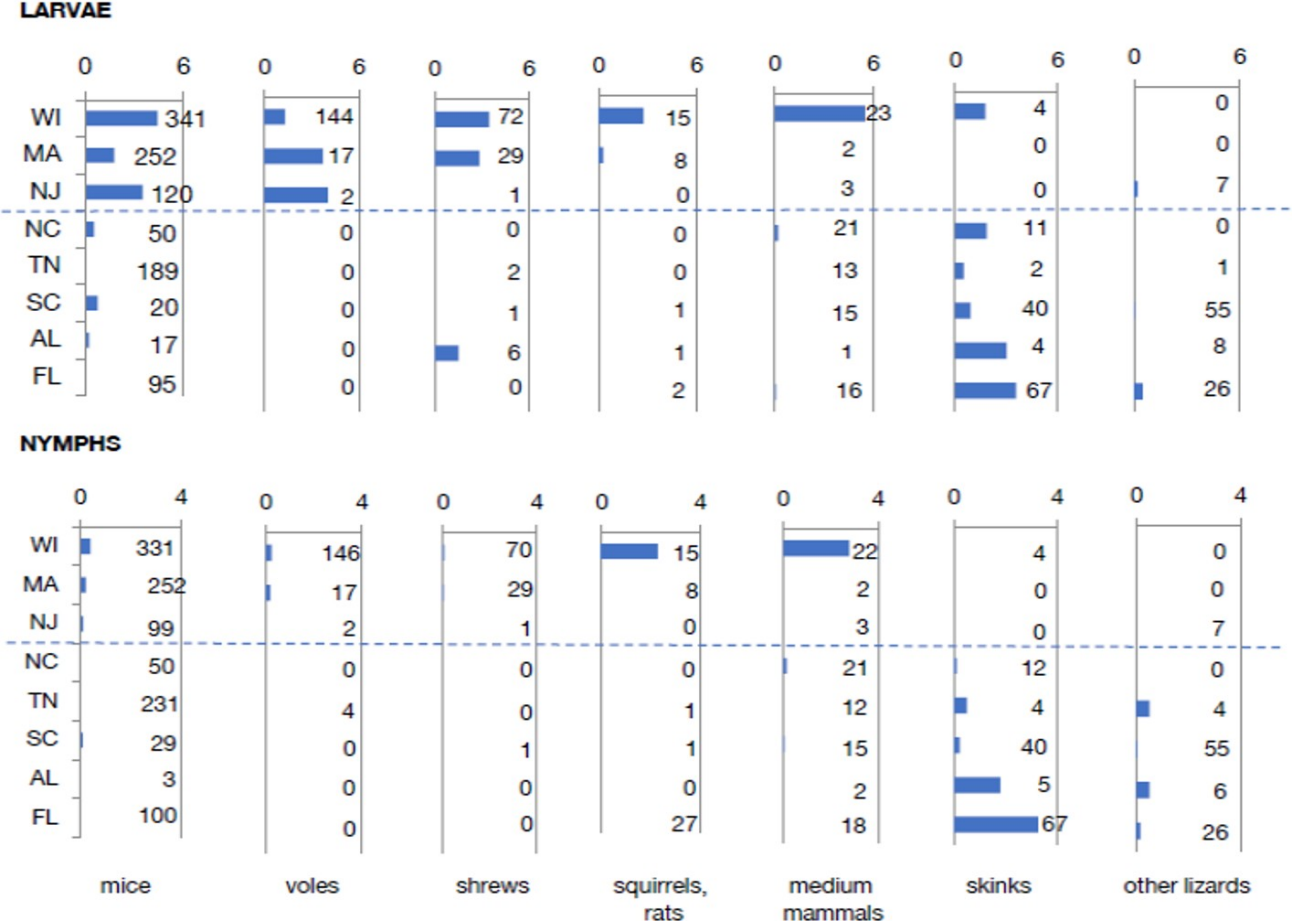
Latitudinal trends in tick numbers on hosts. Mean number of ticks per individual animal in each category over the season. Numbers to the right of bars indicate the number of individual captured animals from which each mean was calculated. Dashed line divides northern from southern sites. Data are available in S8 Data.

Overall abundance of ticks on hosts at a site can be quantified as the mean number of ticks collected from all hosts per sample, over the entire activity season of that tick stage [16]. We used this approach in Fig 2 E–H to quantify overall abundance of ticks at each site. This approach is superior to flag/drag sampling for latitudinal comparisons because north–south differences in tick host-seeking behavior [17] result in differential sampling effectiveness of tick life stages on a latitudinal gradient (S1 Fig). We used multiple vertebrate sampling methods designed to capture a broad variety of ground-dwelling tick host species, and we used a standardized sampling protocol at our sites so the samples are directly comparable. Abundance of both larvae and nymphs showed modest trends of increase with latitude (Fig 2E–2H), but there was a great deal of variability among sites, and the latitudinal trends were statistically significant for larvae but not for nymphs. To assess the combined effects of differences in tick abundance and distributional patterns of ticks on hosts, we quantified the number of ticks per individual host animal in each host category over the season (Fig 5). Here, the bars represent the mean number of ticks collected per animal in each category; the number to the right is the total number of animals used to calculate that mean, and the dashed line divides sites above and below 37° N latitude. Latitudinal trends in the numbers of immature ticks per individual host animal tended to be positive at higher latitudes for small mammals like mice and negative for skinks (Fig 5 and S2 Table). These results clearly document predominant attachment to mammals in the northern sites and to lizards (especially skinks) in the southern sites, with very low levels of attachment to efficient reservoir hosts (such as mice) in the south.

The number of ticks per individual host animal is critical in determining the level of amplification of a pathogen (the spread of the pathogen through vector and host populations), because as the number of ticks that attach to an animal increases, the probability also increases that the host will be exposed to the pathogen and then potentially serve as a reservoir of infection for transmission to uninfected vectors. We assessed the factors that influence the number of ticks that attach to individual mice, the primary reservoirs for *B*. *burgdorferi* infection, at our northern and southern sites by applying general linear models (GLMs) to assess the effects of overall tick abundance, overall host abundance, and host species richness on mean numbers of ticks per individual host animal. GLM analyses to assess log numbers of ticks per mouse at all sites, with “North versus South” as a class variable, found significant differences between northern and southern sites for both larvae (***p*** = 0.0002) and nymphs (***p*** < 0.0001), so we analyzed northern sites separately from southern sites in Table 2. Overall tick abundance (the mean number of ticks collected from all hosts per sample week, Fig 2H) predicted the number of larvae per mouse in the north and larvae per skink in the south, as well as the number of nymphs per skink at the southern sites (Table 2). Also, overall host density was negatively related to the number of larvae per mouse in the north. Host species richness, however, did not contribute significantly in any of the models. Therefore, the factors that determine the number of ticks per host animal were most closely related to tick and host abundance at our sites, not to host diversity.

**Table 2:** Factors affecting the number of ticks per host animal^a^.

Region	Host	Tick abundance	Host abundance	Host species richness	*R*^*2*^
**Larvae**					
North^b^	Mice	0.622***	−0.043***	0.012	0.867***
South^b^	Mice	0.110	−0.002	0.004	0.191
South^b^	Skinks	0.974***	−0.041	0.070	0.704***
**Nymphs**					
North^b^	Mice	0.111	−0.002	0.002	0.408
South	Mice	−0.005*	−0.001	<0.0001	0.472
South^b^	Skinks	0.566***	0.005	−0.004	0.868***

***R***^***2***^ values are for GLMs with “site” as a class variable (to account for environmental differences among sites) plus the variables “tick abundance” (= number of ticks collected from all hosts at the array per sample), “host abundance” (= number of host individuals captured at the array per sample), and “host species richness” (= total number of host species captured at the sample array).

^a^Entries for each variable are model coefficients. Significance for independent variables and for entire model (***R***^***2***^): *p < 0.05, **p < 0.01, ***p < 0.001.

^b^Numbers of ticks log transformed to improve model fit.

GLM, general linear model.

**Fig 6:**
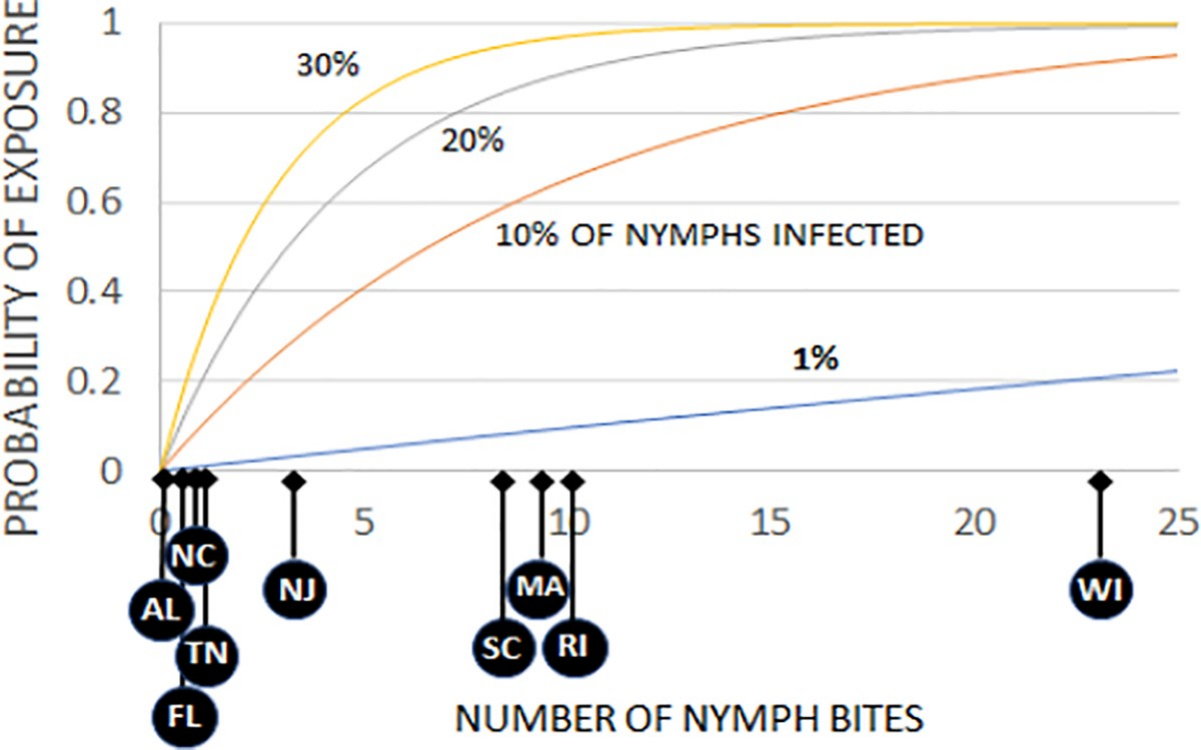
Probability of exposure to spirochetes for mice bitten by varying numbers of nymphal ticks. Probabilities calculated for different levels of infection prevalence in ticks. Approximate yearly numbers of nymphs per mouse over the season at the 8 primary field sites (2011–2012) and at Rhode Island (2012) indicated below horizontal axis. Data are available in S9 Data.

The implications of these patterns of tick–host associations for transmission of Lyme spirochetes can be seen by assessing the probability of a potential reservoir host animal, such as a mouse, to be exposed to infection. The probability of exposure depends on the number of nymphal tick bites and the proportion of ticks that are infected with the spirochete [33,34]. We estimated these probabilities using the binomial probability of exposure at different numbers of tick bites and different levels of infection prevalence in ticks and then compared these probabilities at our study sites (Fig 6). Our study sites are arranged along the horizontal axis of Fig 6 based on rough estimates of the total numbers of tick bites per mouse over the season. Clearly, the northern sites are distributed at high enough levels of the horizontal axis, so that even with relatively low infection prevalence in nymphs, the probability of exposure to the spirochete is high for a mouse. The southern sites are mostly at the low end of the horizontal axis, so that even with moderate infection prevalence in nymphs, the probability that a mouse would be exposed to spirochetes is low. One exception is the South Carolina site, where mice could potentially be exposed to the spirochete if nymphal infection rates were high. The number of ticks per mouse at this site was low (0.1 nymphs per mouse), but the long active season resulted in moderate numbers of ticks per mouse over the year. However, the vast majority of both nymphs and larvae are on skinks and not mice at this site (Fig 3), so infection in ticks remains low. Two of the southern sites, North Carolina and Tennessee, had a large proportion of ticks on mice (partly because skinks were relatively uncommon, Fig 3), but the low tick numbers overall resulted in few ticks per mouse (Figs 2E–2H and 4), lowering the probability of exposure (Fig 6). Thus, low tick densities at some southern sites contributed to the low infection rates and low numbers of host-seeking ticks, resulting in low densities of infected nymphs (DIN), even at southern sites with low lizard abundance.

## Discussion

The dilution effect has been invoked to explain patterns of tick infection with zoonotic pathogens, including the distribution of Lyme disease in the US [6]. Our results indicate that the north–south trend in tick infection prevalence with *B*. *burgdorferi*, and the associated gradient in human Lyme disease, results from selective attachment of ticks to lizards, especially skinks, in the southern states, and not from simple dilution of ticks among host species. Latitudinal trends in tick host-seeking behavior [4] and in tick densities at some sites (Fig 5) also contribute to this north–south gradient.

### North–south patterns in tick–host associations and spirochete infection prevalence

Infection prevalence of *I*. *scapularis* with *B*. *burgdorferi* is substantial in the northern states and low in the south, dropping precipitously below roughly 37° N latitude (Fig 1), a pattern recently reported by other investigators [18]. This pattern does not closely match trends in species diversity of ground-dwelling vertebrates (Fig 2A–2D), which show a gradual latitudinal trend, and thus does not support the hypothesis that simple dilution effects explain the latitudinal trend in tick infection with spirochetes or Lyme disease incidence. We took comprehensive samples of mammals and lizards at our sites, and only occasional bird samples, but the overall latitudinal trend in bird diversity similarly does not support the dilution effect [6]. Furthermore, simple geographical patterns of tick abundance (Fig 2E–2H) do not seem to explain the dramatic drop in spirochete prevalence between northern and southern sites (Fig 1B).

Patterns of actual tick–host associations tell a somewhat more complex story. It is important to understand that larval ticks are not infected with the Lyme spirochete, *B*. *burgdorferi*, when they hatch from the egg [35]. The uninfected larvae attach to host animals, which, if infected, can transmit infection to the larvae. Infected engorged larvae maintain infection when they molt to the nymphal stage. Nymphs are active earlier in the season than larvae in the Northeast [35], and larval and nymphal activity more closely coincide in spring and summer in the northern midwest [15,36]. Therefore, hosts are exposed to infection by nymphs in northern sites at the same time or before uninfected larvae feed on the hosts, resulting in high proportions of larvae acquiring spirochetes if the host species is an efficient reservoir (i.e., it infects a high proportion of feeding larvae) or low proportions if the host is an inefficient reservoir (i.e., it infects none or a low proportion of feeding larvae). The engorged larvae molt into nymphs, which can then transmit the infection to other hosts, including people [35]. Thus, the distribution of larvae on hosts affects the proportion of nymphs that are infected, and this pattern does not follow a simple latitudinal gradient in eastern North America.

### Dilution versus nonrandom host selection

The results in Figs 3–5 suggest selective attachment of ticks to different categories of hosts and that this attachment pattern differs from north to south. If ticks were simply being diluted among hosts, then the proportions of hosts from each category, and the proportions of ticks collected from each category, would show the same relationship at all sites, and the percent similarity of the distributions of hosts and ticks among host categories would not change with latitude. However, the relationship of host and tick distributions clearly changes with latitude (Fig 3), producing a latitudinal pattern in percent similarities of distributions of hosts among categories compared to ticks attaching to those host categories (Fig 4C and 4D). These results indicate a latitudinal gradient in host selection by *I*. *scapularis*, rather than a simple gradient in dilution of ticks among hosts.

Whether this selective attachment results from behavioral factors (e.g., host preferences) or ecological factors (e.g., differences between times or sites of tick and host activity) is not clear. It is possible, for example, that northern ticks might be host generalists (attaching largely to the abundant rodents), while the southern ticks might be specialists (attaching predominantly to skinks). One behavioral study, however, found no consistent difference in attachment of southern *I*. *scapularis* to mice versus lizards in the lab [37]. The differential questing behavior of southern compared to northern ticks [4,17] might contribute to this differential attachment pattern because southern ticks remain below the leaf litter surface to seek hosts while northern ticks seek hosts atop the leaf litter, but additional studies examining the determinants of host-seeking behavior are needed.

### Ecological determinants of tick infection prevalence

Our results show that the decline in tick infection prevalence from north to south results from a combination of lower tick densities at some southern sites (e.g., our sites in Tennessee and North Carolina) and a broad shift in host associations with latitude. Northern larval and nymphal ticks are generally abundant on hosts (Fig 5), including efficient reservoir hosts such as mice, voles, and shrews (Table 1). Southern ticks tend to be uncommon on reservoir hosts, and more common on lizards, particularly skinks (Figs 3 and 5), which are relatively poor reservoirs for the Lyme spirochete. Two southern sites where skinks were relatively uncommon (Tennessee and North Carolina) had very low tick populations, and thus few ticks on mice (Fig 5). One southern site had substantial tick populations (Florida), but the larval and nymphal ticks displayed strong selective attachment to skinks and not to mice (Fig 5). The relative rarity of ticks attaching to reservoir hosts in the south results in low levels of infection of host-seeking ticks with Lyme spirochetes (Fig 1B).

The other major factor that affects human exposure to Lyme spirochetes along a geographical gradient is that nymphal ticks, the primary vector stage of Lyme spirochetes to humans [35], differ in host-seeking patterns in northern versus southern locales; the northern ticks often climb to the top of the leaf litter and quest on leaf tops and twigs to seek hosts, while southern ticks tend to remain down in the leaf litter beneath the surface [17]. Therefore, a summer walk through the woods in the north results in direct exposure to host-seeking *Ixodes* ticks, while a similar walk in the south does not. The tendency to quest high (at or above the leaf litter surface) is well correlated with incidence of human Lyme disease [4], likely accounting for the far greater numbers of tick bites by this species in northern compared to southern locales [5]. This difference in host-seeking behavior might result from climatic factors because the warmer southern temperatures result in desiccation stress above the leaf litter, possibly providing a selective pressure for southern ticks to remain down below the surface [38].

Therefore, our findings support the hypothesis that 2 ecological and behavioral factors explain the relative rarity of Lyme disease in the southern US. First, people are bitten by fewer nymphal *I*. *scapularis* in the south [5] because southern nymphs seek hosts below the leaf litter (where they generally do not encounter humans), while northern nymphs abundantly seek hosts on top of the leaf litter [4,17] where they can frequently encounter people. Second, even when people are bitten in the south, the ticks have a considerably lower infection prevalence with *B*. *burgdorferi* than in the north (Fig 1B), because few of the southern ticks attach to reservoir host species, either because ticks are rare at some sites or because they selectively attach to hosts that are poor reservoirs, such as skinks (Fig 5). Our results provide ecological mechanisms that are consistent with geographical genetic gradients in *I*. *scapularis* populations [18].

These results provoke interesting questions about the future distribution of Lyme disease, especially in view of climate change. Tick population and spirochete transmission models suggest that the distribution of *I*. *scapularis* will expand northward, as will human cases of Lyme disease [39]. Indeed, northward expansion of ticks and Lyme disease in southern Canada is already evident [40]. However, the southern edge of the range presents complex problems. Will ticks in the mid-Atlantic states start to behave more like southern ticks, resulting in less Lyme disease in Virginia and Maryland? This might be expected if populations of skinks increase with climate change in the mid-Atlantic states and if higher temperatures result in selective pressure on mid-Atlantic ticks to seek hosts below the leaf litter surface to avoid desiccating conditions produced by the increased temperatures [38]. However, the situation is complicated by the history of *I*. *scapularis* in North America, which has been characterized by range expansions from refugia in the northeastern coastal states and northern midwestern sites [41]. Indeed, recent studies have identified expansion of northern-type ticks south along the Appalachian Mountains of southwestern Virginia [42] and possibly in river valleys in northeastern Tennessee [43]. These ticks might act like northern ticks, resulting in an initial increase in the incidence of Lyme disease in the south. Indeed, Lyme disease incidence has increased in recent years in southwestern Virginia [42], and increases in canine prevalence of anti-*B*. *burgdorferi* antibodies also have been observed on both sides of the Appalachians in these areas [44]. However, it is plausible that, with time, these ticks will undergo selection to act more like southern ticks, resulting in lowering incidence of Lyme disease. Indeed, *I*. *scapularis* might disappear altogether from the most southern limits of its range due to inhospitable climate for both ticks and hosts. At present, we cannot predict whether human cases of Lyme disease will increase in some southern states or whether Lyme disease will decline in the mid-Atlantic states with increasing lizard populations and with climate change–related temperature increases. Given the results of our study, we now know, at least, what questions need to be answered in order to make that prediction.

## Materials and methods

### Sampling program

Nine sample sites were located in the eastern and central US, from north to south (Fig 1). The sites (with approximate latitude/longitude) included Fort McCoy, Wisconsin (44.04 N, -90.68 W), Cape Cod National Seashore, Massachusetts (41.87 N, -69.98 W), scattered sites in Rhode Island (41.41 N, -71.62 W) and in the pine barrens of central New Jersey (39.85 N, -74.57 W), Mattamuskeet National Wildlife Refuge, North Carolina (35.48 N, -76.31 W), Arnold Air Force Base, Tennessee (35.33 N, -86.10 W), Savannah River Site, South Carolina (33.29 N, -81.73 W), Oakmulgee Talladega National Forest, Alabama (32.96 N, -87.46 W), and Tall Timbers Research Station, Florida (30.66 N, -84.21 W). Four sites were sampled from 2010 through 2012 (Wisconsin, Massachusetts, Tennessee, and South Carolina), 4 were sampled in 2011 and 2012 (New Jersey, North Carolina, Alabama, and Florida), and 1 was a partial sample in 2012 (Rhode Island).

Each site had 2 to 3 sampling arrays, spaced >3-km apart (to minimize movement of hosts among arrays). Each array (S2 Fig) was approximately 1 ha, including a 7 × 7 grid of Sherman collapsible live traps (23 × 7.6 × 9 cm) placed 15-m apart (Sherman Traps, Tallahassee, Florida, US), 4 pitfall trap arrays (1 at each edge), 4 Tomahawk traps (81.3 × 25.4 × 30.5 cm), 1 set at each edge of the array (Tomahawk Live Trap, Hazelhurst, Wisconsin, US), 20 pairs of plywood and corrugated metal cover boards (each 0.6 × 0.6 m), 20 burlap skirts on trees (1-m^2^ at breast height), 4 action-activated wildlife cameras (Bushnell Trophy Cam, model #119405, Bushnell, Overland Park, Kansas, US), 1 scent-baited camera at each corner of the array, and a centrally located weather station (HOBO Pro v2 data loggers, Onset Computer, Bourne, Massachusetts, US), which recorded temperature and relative humidity hourly (1 data logger at the leaf litter surface and 1 at 0.5-m height). The Sherman traps were baited with crimped oats, and the Tomahawk traps were baited with canned sardines. The pitfall trap arrays each consisted of five 5-gal plastic buckets (with drainage holes) sunk to ground level with aluminum drift fences placed in a cross pattern, with buckets at the end of each 10-m arm and at the center. Sites were sampled every other week during tick activity periods in 2010 and every third week in 2011 and 2012. Each sample week included 2 nights of trapping, 1 check of cover boards and burlap skirts, and 1 set of 8 flag/drag samples (see references [14–16] for details). The sampling program was identical at all sites, except at the North Carolina site, where pitfall traps could not be constructed because of physical conditions, and the Rhode Island site, where only flag/drag and Sherman trap samples were taken.

Host-seeking ticks were collected from 8 flag/drag transects (90 m each, using 1-m^2^ white flannel flags and drags), which were taken at the edges and between each row of Sherman traps in each array (total of 720 m per sample at each array). Investigators stopped every 15 m on each transect, the numbers of ticks were counted, and up to 10 specimens were collected and placed in 95% ethanol (the rest were released). Captured animals were mostly examined without anesthesia, except for some medium-sized mammals, such as raccoons, which were anesthetized using standard methods [15,45]. Routine data were taken (species, sex, age, weight, etc.), and each animal was examined for attached ticks, starting at the head and working backward, for a maximum examination time of 5 minutes. Animals were then released (after recovery for anesthetized individuals) at the point of capture.

### Estimation methods

North–south trends in infection prevalence were assessed using logistic regression (SAS, version 9.4, LOGISTIC procedure). Host diversity parameters (Fig 2A–2D) were calculated using all of the vertebrate host species sampled at each array at each site. Estimates of tick and host numbers (for each tick stage) were taken from the first day to the last day a tick of that stage was collected at that site by any sampling method (S1 Table), because tick phenologies differed from site to site [15]. Species diversity was assessed for each sampling array using the Shannon–Wiener Index, H = − ∑ *p*_*i*_ log *p*_*i*_, where *p*_*i*_ is the proportion of the numbers of species *i* in the samples. Estimates of total species richness from trap data for each entire site were calculated using the SPECRICH estimation program (https://www.mbr-pwrc.usgs.gov/software/specrich.html), written by J.E. Hines, based on Burnham and Overton [46]. Regressions of species diversity measures with latitude were performed using SAS, version 9.4 (SAS Institute, Cary, North Carolina, US), GLM procedure. Evenness was calculated as 1 minus the Berger–Parker Index = 1 − proportion of the total catch that was the most abundant species.

Hosts were divided into categories based roughly on size and on previous literature estimates of importance as reservoirs. Mice, voles, and shrews have generally been considered important reservoir hosts for *B*. *burgdorferi* [10,11,13,21–26,47] and generally demonstrate high reservoir competence (Table 1). Squirrels and rats are slightly larger, vary in reservoir competence (Table 1), and have been identified as important reservoirs only in occasional local studies [28,48]. Medium mammals (at least, those in our samples) display low reservoir competence (Table 1) and so are generally not considered important reservoirs. Blacklegged ticks readily attach to skinks but attach relatively rarely to fence lizards and anoles [12,32] and rarely attach to snakes.

Logistic regressions on the change in proportion of ticks from each host category with latitude were performed with SAS, version 9.4, LOGISTIC procedure. The dependent variable was the number of ticks collected from hosts in each category divided by the total number of ticks collected from all hosts. Percent similarity of hosts in each category, and ticks collected from hosts in each category (Fig 3C), were calculated using the following formula [45], % Similarity = 100 − 50**∑**│***p***_***a*,*i***_−***p***_***b*,*i***_│, where ***p***_***a*,*i***_ = proportion of host individuals ***a*** in category ***i*** and ***p***_***b*,*i***_ = proportion of ticks ***b*** from hosts in category ***i***.

Numbers of ticks from animals were compiled at each site (Figs 2–5), and tick abundance on hosts was calculated as the mean of the total numbers of ticks collected from all hosts during each sample week, averaged over the season. For these estimates, we used hosts collected on the first trapping day each sample week, to avoid including data from animals from which the ticks had been removed earlier in the week.

We estimated the total number of ticks per mouse over the season (Fig 6) by multiplying the mean number of ticks per mouse over the season by twice the number of weeks in the season (because a nymph typically stays attached 3 to 4 days, so there are roughly 2 cohorts of nymphs on a mouse per week). We then multiplied by a factor of 1.2 to account for the ticks on the mouse that we missed with the visual inspection [49]. The probability of exposure to the pathogen (***P***_***e***_) was estimated as the binomial probability of being bitten by at least 1 infected tick [34], given ***n*** tick bites, and prevalence of infection of ***k***_***v***_ in ticks: ***P***_***e***_ = 1 –(1 –***k***_***v***_)^***n***^.

### Infection testing

Ticks were returned to the lab in 95% ethanol and tested using previously published methods [50]. Briefly, total genomic DNA was extracted from each tick (Qiagen, Valencia, California, US), which was then subject to quantitative PCR with a probe for *B*. *burgdorferi* 16s rDNA. The forward primer, at 900 nM, was 5′-CGTGTAAACGATGCACACTTGGT, and the reverse primer was 5′-GGCGGCACACTTAACACGTTAG. The dye-labeled probe, at 200 nM, was 6FAM-TTCGGTACTAACTTTTAGTTAA, with a minor groove binding (MGB) protein (Applied Biosystems, Foster City, California, US). The thermal cycler was set for 50°C for 2 minutes, followed by 45 cycles of 95°C for 15 seconds and 63°C for 60 seconds. Each 96-well plate included negative extraction controls, negative PCR controls (PCR water), positive PCR controls, and the specimens for testing.

### Statistical analyses

Regression analyses were carried out using the GLM procedure in SAS, version 9.4. Overall analyses of species diversity measures with latitude used arrays as replicates at each latitude. For species richness, we estimated the total number of species at each site using SPECRICH. We also carried out finer-tuned analyses of trends of species diversity, species richness, and evenness of mammals and lizards at each sampling array with latitude separately in 2011 and 2012 and specifically during the time of year when larvae and nymphs were active (S1 Table). GLM analyses to assess log numbers of ticks per mouse at all sites, with “North versus South” as a class variable, found significant differences between northern and southern sites for both larvae (***p*** = 0.0002) and nymphs (***p*** < 0.0001), so we analyzed northern sites separately from southern sites in Table 2. GLMs that analyzed the roles of various factors affecting the numbers of ticks per mouse or per skink (Table 2) all used “site” as a class variable (to account for different environmental conditions among sites) and used tick abundance (the mean number of ticks collected from all hosts per sample over the season), host abundance (the mean number of host animals captured per sample over the season), and host species richness (the total number of host species collected at each site over the entire project) as independent variables. The effect of year was not significant in any of these analyses, so “year” was not included in the final models. There were no significant interactions among the metric variables in these analyses, so the results from the models with only main effects are presented in Table 2.

### Ethics statement

Animal handling protocols received Institutional Animal Care and Use Committee (IACUC) approvals from Michigan State University (protocol 06/09-094-00), the University of Rhode Island (protocol AN09-04-016), Rutgers University (protocol 12–021), Hofstra University (protocols 08/09-7, 10/11-8, 11/12-9), the University of Tennessee (protocol 1846–0512), Georgia Southern University (protocols I09011, I11004), and Patuxent Wildlife Research Center. Collecting and research permits were obtained at each site and at state and federal levels (when required for vertebrate samples) and are available on request.

## Supporting information

S1 Table Dates of first and last appearances of larval and nymphal *Ixodes scapularis* in samples (including both flag/drag samples and samples from hosts). (DOCX)

S2 Table Relationships of mean numbers of ticks per host animal with latitude. (DOCX)

S1 Fig The numbers of nymphal *Ixodes scapularis* divided by the numbers of adults captured in flag/drag samples at our sample sites. The lower ratios of nymphs/adults at the southern sites demonstrate the difficulty in collecting nymphs using flag/drag samples in the south. Data are available in S2 Data.
(TIFF)

S2 Fig Field sampling array. Each major sample site had 2 or 3 sampling arrays for tick hosts, ticks from hosts, and host-seeking ticks. Dashed lines are flag/drag transects.
(TIFF)

S1 Data Infection of adult *Ixodes scapularis* collected by flag/drag sampling. Samples taken from 9 sites. AEDC, Arnold Air Force Base, Tennessee; CACO, Cape Cod National Seashore, Massachusetts; FTM, Fort McCoy, Wisconsin; MNWR, Mattamuskeet National Wildlife Refuge, North Carolina; NJ, scattered oak/pine barrens sites in central New Jersey; OTNF, Oakmulgee Talladega National Forest, Alabama; SRS, Savannah River Site, South Carolina; Rhode Island, scattered sites in southern Rhode Island; TTRS, Tall Timbers Research Station, Florida. Binomial 95% confidence intervals calculated using the estimator at the website http://vassarstats.net/prop1.html based on [51,52].
(XLSX)

S2 Data *Ixodes scapularis* collected by flag/drag sampling. “Best estimate” based on field identifications and counts corrected by morphological identifications in lab (database date: 7 March 2017).
(XLSX)

S3 Data *Ixodes scapularis* collected from hosts. “Best estimate” based on field identifications and counts corrected by morphological identifications in lab (database date: 7 March 2017). Raw data and species codes are provided, along with estimates of diversity calculated as detailed in the Materials and methods section.
(XLSX)

S4 Data Tick densities on hosts in each array at each site in 2011 and 2012. Means are average numbers of ticks collected from all hosts per sample.
(XLSX)

S5 Data Proportions of hosts in each host category and proportions of ticks collected from hosts in each category, 2011 and 2012. (XLSX)

S6 Data Results of logistic regressions of proportions of ticks from each host category with latitude. OR with 95% confidence limits for latitudinal trends in proportion from each host category. OR, odds ratio.
(XLSX)

S7 Data Percent similarity of proportional distribution of hosts collected in each category with proportional distribution of ticks collected from hosts in each category at each site. (XLSX)

S8 Data Number of ticks per animal sampled in each host category, with numbers of host animals used for each calculation. (XLSX)

S9 Data Probability of exposure to pathogen at various numbers of tick bites at several levels of pathogen prevalence in ticks. Estimated total numbers of ticks per mouse over the season at each site based on data from 2011 and 2012. See Materials and methods section for calculation methods.
(XLSX)

## Abbreviations

DIN: densities of infected nymphs
GLM: general linear model
IACUC: Institutional Animal Care and Use Committee
MGB: minor groove binding
