# Correction: Why Lyme disease is common in the northern US, but rare in the south: The roles of host choice, host-seeking behavior, and tick density

**DOI:** 10.1371/journal.pbio.3001396

**Published:** 2021-09-08

**Authors:** Howard S. Ginsberg, Graham J. Hickling, Russell L. Burke, Nicholas H. Ogden, Lorenza Beati, Roger A. LeBrun, Isis M. Arsnoe, Richard Gerhold, Seungeun Han, Kaetlyn Jackson, Lauren Maestas, Teresa Moody, Genevieve Pang, Breann Ross, Eric L. Rulison, Jean I. Tsao

In the Estimation methods subsection of the Materials and methods, there is an error in the third sentence of the third paragraph. The correct sentence is: Percent similarity of hosts in each category, and ticks collected from hosts in each category (Fig 4C and 4D), were calculated using the following formula 45], % Similarity = 100 − 50**∑**|***p***_***a,i***_−***p***_***b,i***_|, where ***p***_***a,i***_ = proportion of host individuals ***a*** in category ***i*** and ***p***_***b,i***_ = proportion of ticks ***b*** from hosts in category ***i***.

There is a formatting error in Fig 3. Additionally, in Fig 5, the number of individuals from the category "squirrels, rats" that were sampled in Florida to determine the numbers of larvae should be 27.

**Fig 3:**
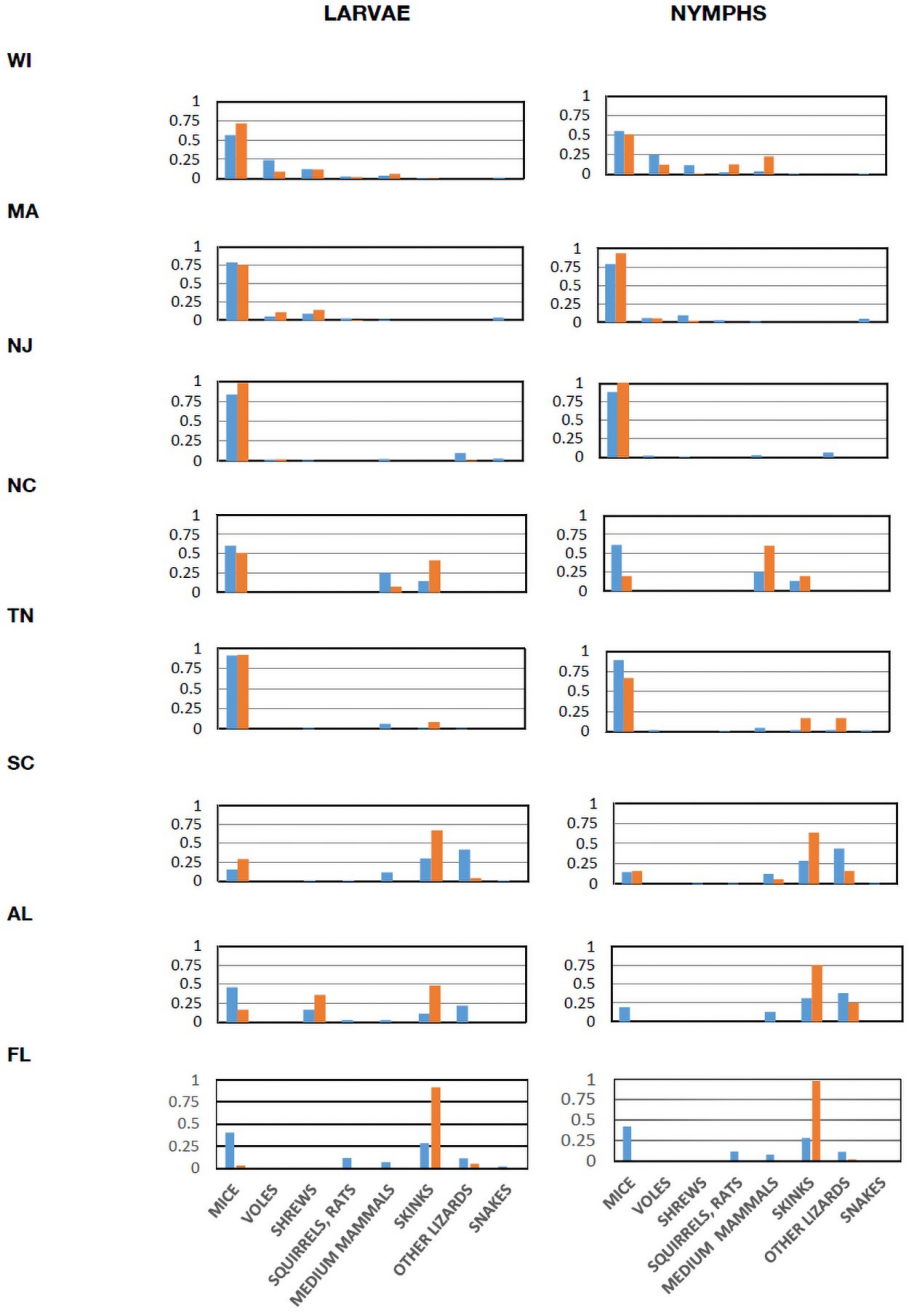
Proportions of hosts of different categories in samples and proportions of ticks collected from hosts in each category. Blue bars are proportions of hosts in each category, and orange bars are proportions of ticks collected from hosts in each category. Taxa in each category: mice (*Peromyscus* and *Ochrotomys*), voles (*Myodes* and *Microtus*), shrews (*Sorex* and *Blarina*), squirrels, rats (*Tamias*, *Glaucomys*, *Tamiasciurus*, *Neotoma*, and *Oryzomys*), medium mammals (*Procyon* and *Didelphis*), skinks (*Plestiodon* and *Scincella*), other lizards (*Sceloporus* and *Anolis*), and snakes (*Diadophis*, *Storeria*, *Thamnophis*, and *Coluber*). Data combined from 2011 and 2012 (when all 8 sites were sampled). Data are available in S5 Data (raw data in S3 Data).

**Fig 5:**
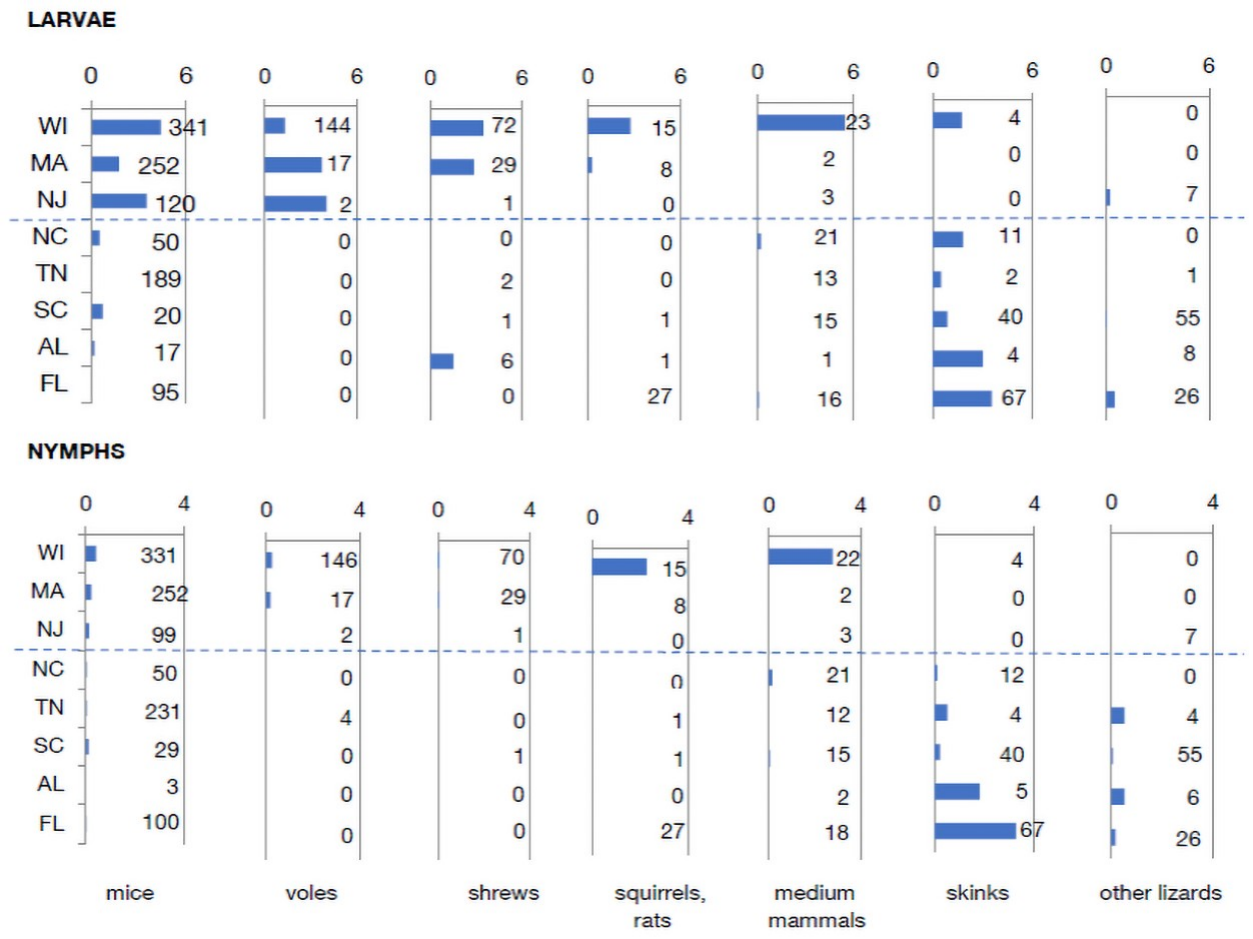
Latitudinal trends in tick numbers on hosts. Mean number of ticks per individual animal in each category over the season. Numbers to the right of bars indicate the number of individual captured animals from which each mean was calculated. Dashed line divides northern from southern sites. Data are available in S8 Data.
