# Supplementary material for: Why Lyme disease is common in the northern US, but rare in the south: The roles of host choice, host-seeking behavior, and tick density
Source: PLoS Biol. 2021 Jan 28;19(1):e3001066. doi: 10.1371/journal.pbio.3001066 (PMC7842935; doi:10.1371/journal.pbio.3001066)
Supplement: S1 Table — (DOCX) [file pbio.3001066.s001.docx]

**S1 Table. Dates of first and last appearances of larval and nymphal *Ixodes scapularis* in samples (including both flag/drag samples and samples from hosts)**

|  |  | **Larvae** |  | **Nymphs** |  |
| --- | --- | --- | --- | --- | --- |
| **Site** | **Year** | **first** | **last** | **first** | **last** |
|  |  |  |  |  |  |
| WI | 2010 | 5/25 | 10/24 | 5/25 | 10/24 |
|  | 2011 | 5/24 | 10/23 | 5/6 | 10/2 |
|  | 2012 | 5/5 | 10/9 | 4/22 | 10/8 |
| MA | 2010 | 6/2 | 10/20 | 5/20 | 11/23 |
|  | 2011 | 6/1 | 10/5 | 5/6 | 9/14 |
|  | 2012 | 5/31 | 9/14 | 5/31 | 9/13 |
| RI | 2012 | 5/24 | 10/21 | 5/24 | 10/17 |
| NJ | 2011 | 6/29 | 10/20 | 5/25 | 9/21 |
|  | 2012 | 7/12 | 10/24 | 5/16 | 6/8 |
| NC | 2011 | 6/30 | 11/16 | 6/30 | 11/16 |
|  | 2012 | 4/12 | 8/12 | 4/13 | 8/11 |
| TN | 2010 | 6/23 | 9/19 | 5/24 | 8/18 |
|  | 2011 | 7/12 | 8/8 | 4/12 | 7/12 |
|  | 2012 | 4/15 | 9/9 | 4/15 | 8/12 |
| SC | 2010 | 4/29 | 10/9 | 4/29 | 9/10 |
|  | 2011 | 2/6 | 7/29 | 1/8 | 11/19 |
|  | 2012 | 5/3 | 7/27 | 3/8 | 8/23 |
| AL | 2011 | 5/21 | 6/14 | 3/13 | 6/14 |
|  | 2012 | 4/22 | 7/15 | 3/31 | 3/31 |
| FL | 2011 | 5/15 | 9/21 | 4/27 | 9/24 |
|  | 2012 | 2/17 | 8/15 | 2/29 | 9/10 |
