# Supplementary material for: Why Lyme disease is common in the northern US, but rare in the south: The roles of host choice, host-seeking behavior, and tick density
Source: PLoS Biol. 2021 Jan 28;19(1):e3001066. doi: 10.1371/journal.pbio.3001066 (PMC7842935; doi:10.1371/journal.pbio.3001066)
Supplement: S2 Table — (DOCX) [file pbio.3001066.s002.docx]

**S2 Table. Relationships of mean numbers of ticks per host animal with latitude**

| **Tick stage** | **Host category** | **Slope** | ***R^2^*** | ***p*** |
| --- | --- | --- | --- | --- |
|  |  |  |  |  |
| Larvae | mice | 0.309 | 0.738 | 0.006 |
|  | voles | 0.264 | 0.501 | 0.0496 |
|  | shrews | 0.222 | 0.548 | 0.057 |
|  | squirrels, rats | 0.140 | 0.467 | 0.062 |
|  | medium mammals | 0.260 | 0.397 | 0.094 |
|  | skinks | -0.163 | 0.326 | 0.140 |
|  | other lizards | -0.016 | 0.177 | 0.299 |
|  |  |  |  |  |
| Nymphs | mice | 0.027 | 0.777 | 0.004 |
|  | voles | 0.017 | 0.707 | 0.009 |
|  | shrews | 0.003 | 0.711 | 0.017 |
|  | squirrels, rats | 0.109 | 0.404 | 0.090 |
|  | medium mammals | 0.131 | 0.399 | 0.093 |
|  | skinks | -0.180 | 0.500 | 0.0497 |
|  | other lizards | -0.023 | 0.234 | 0.225 |
