# Supplementary figures and images for: Why Lyme disease is common in the northern US, but rare in the south: The roles of host choice, host-seeking behavior, and tick density
Source: PLoS Biol. 2021 Jan 28;19(1):e3001066. doi: 10.1371/journal.pbio.3001066 (PMC7842935; doi:10.1371/journal.pbio.3001066)

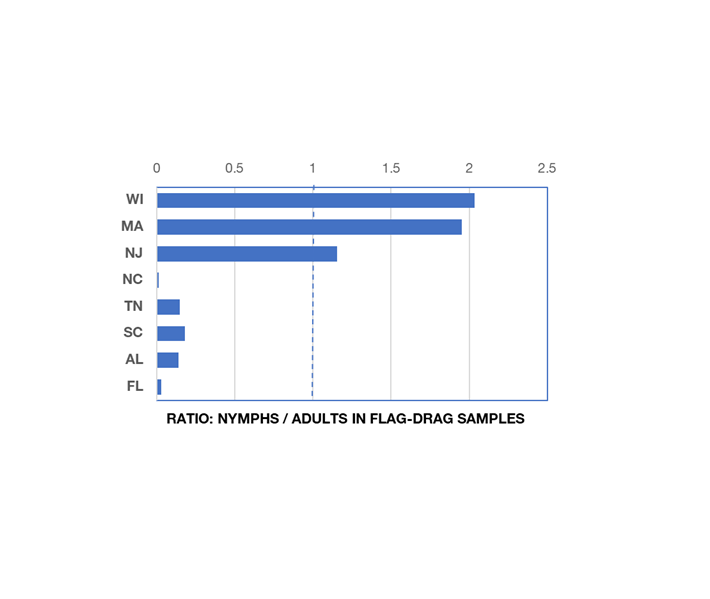

Supplement: S1 Fig — The lower ratios of nymphs/adults at the southern sites demonstrate the difficulty in collecting nymphs using flag/drag samples in the south. Data are available in S2 Data. (TIFF) [file pbio.3001066.s003.tiff]

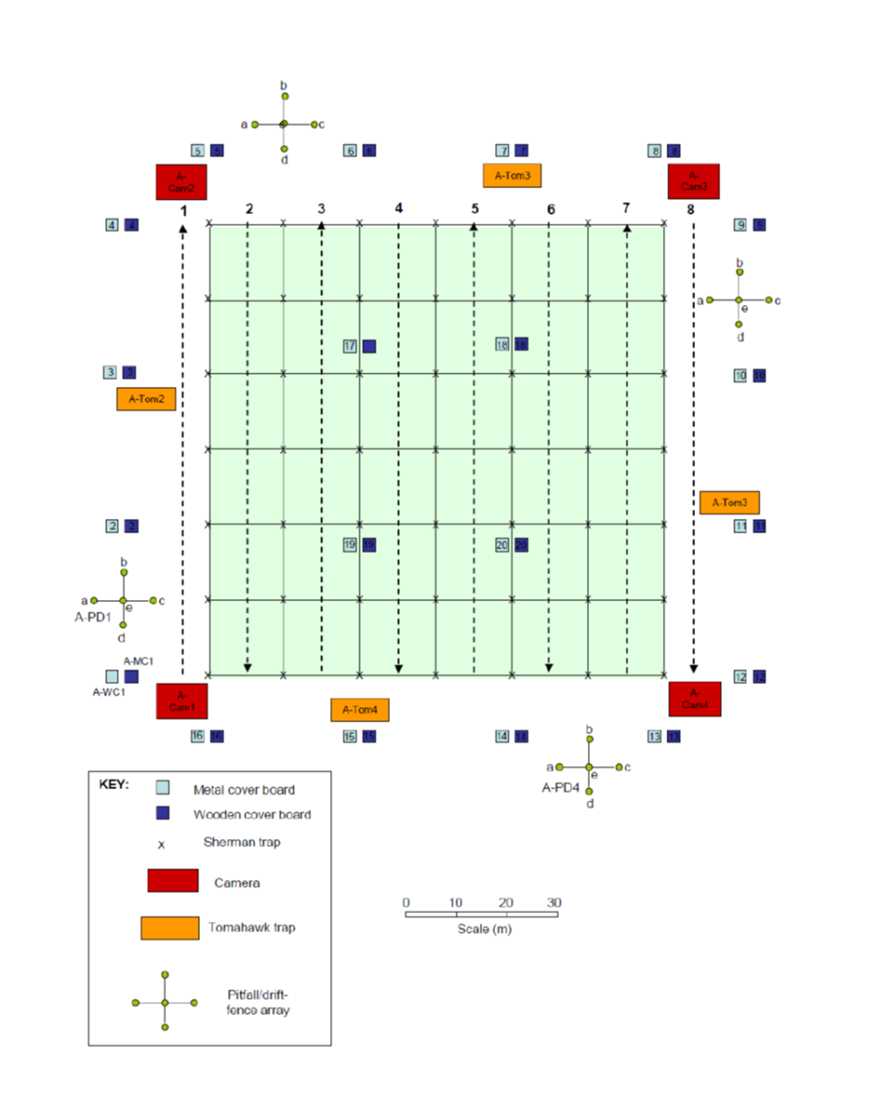

Supplement: S2 Fig — Each major sample site had 2 or 3 sampling arrays for tick hosts, ticks from hosts, and host-seeking ticks. Dashed lines are flag/drag transects. (TIFF) [file pbio.3001066.s004.tiff]
